# Porcine circovirus type 3: immunohistochemical detection in lesions of naturally affected piglets

**DOI:** 10.3389/fvets.2023.1174718

**Published:** 2023-05-04

**Authors:** Franciéli Adriane Molossi, Bruno Albuquerque de Almeida, Bianca Santana de Cecco, Caroline Pissetti, Lauren Ventura, Luciano Brandalise, Gustavo Simão, Fabio Vanucci, Tatiane Terumi Negrao Watababe, Itabajara da Silva Vaz Jr., David Driemeier

**Affiliations:** ^1^Faculdade de Veterinária, Universidade Federal do Rio Grande do Sul, Porto Alegre, Brazil; ^2^Department of Pathobiological Sciences, Louisiana State University, Baton Rouge, LA, United States; ^3^Centro de Diagnóstico de Sanidade Animal (CEDISA), Concórdia, Brazil; ^4^Agroceres Pic, Rio Claro, Brazil; ^5^Veterinary Diagnostic Laboratory, University of Minnesota, St. Paul, MN, United States; ^6^Department of Population Health and Pathobiology, College of Veterinary Medicine, North Carolina State University, Los Angeles, NC, United States; ^7^Centro de Biotecnologia, Universidade Federal do Rio Grande do Sul, Porto Alegre, Brazil; ^8^Instituto Nacional de Ciência e Tecnologia - Entomologia Molecular, Rio de Janeiro, Brazil

**Keywords:** diagnosis, pathology, PCV3, piglets, immunohistochemistry

## Abstract

This study aimed to evaluate the relationship between porcine circovirus type 3 (PCV3) viral load and histopathological findings in perinatal piglet tissues and to develop an immunohistochemical method for detecting the virus in lesions. The quantitative polymerase chain reaction (qPCR) cycle threshold (Ct) when amplifying PCV3 DNA and the area of perivascular inflammatory infiltrates in different organs [central nervous system (CNS), lung, heart, liver, spleen, and lymph nodes] were compared. To develop an immunohistochemistry technique, rabbit sera were produced against PCV3-capsid protein peptides selected using bioinformatic analyses. The assay was initially implemented using a tissue sample previously tested using qPCR and *in situ* hybridization to optimize the procedure and reagent dilutions. To evaluate immunohistochemistry performance, tissue samples from another 17 cases were analyzed using standardized parameters. The most common microscopic lesion was multisystemic periarteritis, with associated vasculitis, as the mesenteric vascular plexus is one of the most affected organs. Other tissues, such as the heart, lung, CNS, and skeletal muscle, were also affected. Comparison of the Ct values for different tissues showed no significant difference, except in lymphoid organs (spleen and lymph nodes), which had significantly higher viral loads than the CNS tissues. There was no correlation between Ct values and perivascular inflammatory infiltrates. PCV3 immunohistochemistry revealed granular immunolabeling, mainly in the cytoplasm of cells in the vascular mesenteric plexus, heart, lung, kidney, and spleen.

## 1. Introduction

Porcine circovirus type 3 (PCV3) is a small, non-enveloped virus with a circular, single-stranded DNA genome belonging to the *Circoviridae* family (1). PCV3 infection is associated with dyspnea, weakness, and thrown-back ears (2). PCV3 has recently been shown to cause abortion (3–5).

Diagnosis of PCV3-associated clinical disease is challenging because detection of viral DNA by PCR is insufficient, as the virus can be detected in the tissues of apparently healthy pigs (6). Thus, to accurately diagnose the disease caused by PCV3, the gold standard is to evaluate the clinical signs of disease, gross and microscopic lesions, and PCR test results, and perform a confirmatory test, which is usually *in situ* hybridization (ISH), as viral mRNA has been detected within lesions by this method (2–5, 7). However, ISH for PCV3 is expensive and is not available in veterinary pathology laboratories in many countries.

As an alternative to ISH, immunohistochemistry (IHC) could be used; however, standardized protocols and reagents are required. IHC is a valuable diagnostic tool for detecting antigens in affected tissues (8). Detection of the virus in pig tissues by IHC requires high-quality and specific antisera (9). However, until now, no commercially available serum has been developed for the identification of PCV3 in paraffin-embedded pig tissues. An interesting strategy to obtain a highly specific antiserum is to identify species-specific epitopes by analyzing the predicted protein sequences through bioinformatic analyses. Once such epitopes are identified, synthetic peptides can be produced. Using serum directed against a short amino acid sequence can increase the specificity by preventing detection of cross-reactive epitopes from other pathogens.

Therefore, this study aimed to describe the microscopic lesions in PCV3-associated disease and their association with viral load in perinatal piglets and to develop an IHC technique using sera directed against peptides of the PCV3-capsid protein.

## 2. Materials and methods

### 2.1. Case selection

Cases were selected from an established database (2) and included 17 total piglets; 16 3–5-day-old piglets and one 60-day-old piglet, which were diagnosed with PCV3 infection based on clinical and pathological features and conventional PCR; six cases were confirmed by ISH. All piglets showed evidence of thrown-back ears, weakness, and respiratory distress. The 60-day-old piglet also showed a marked decrease in growth rate. This published study did not require to be submitted to the ethics committee because it was a retrospective study that only involved the use of already dead animals, in accordance with the terms of Brazilian Federal Law No. 11,794/08. Also, the owners of the pigs were aware and authorized the carrying out of necropsies, histopathology and complementary exams to elucidate the cause of mortality.

Formalin-fixed paraffin-embedded (FFPE) tissue samples from the central nervous system (CNS), lung, heart, liver, kidney, spleen, tonsils, mesenteric lymph nodes, mesenteric plexus, small intestine, large intestine, stomach, esophagus, urinary bladder, adrenal gland, skin, ears, skeletal muscle (tongue and semimembranosus/semitendinosus), testis, and pampiniform plexus of the 17 selected cases were prepared during routine examination, cut into 3 mm thick sections and stained with hematoxylin and eosin for histological evaluation. Each tissue sample was evaluated under an optical microscope, and the lesions were described individually.

### 2.2. Quantitative PCR

Samples were collected from the CNS, including the frontal cortex, cerebellum, pons, midbrain, and cervical spinal cord, as well as the lung, heart, liver, and spleen/lymph nodes of each piglet and subjected to qPCR. Viral DNA was extracted from the tissue samples using in automated extraction system (IndiMag 48 s; Indical Bioscience) and a commercial kit (IndiMag Pathogen Kit, Indical Bioscience). PCV3 was detected by targeting the conserved *rep* gene with the following primers: PCV3_535_F (5′-TGA CGG AGA CGT CGG GAA AT-3′) and PCV3_465_R (5′-CGG TTT ACC CAA CCC CAT CA-3′) and probe PCV3_535_F (5′-TGA CGG AGA CGT CGG GAA AT-3′), as described previously (10). For qPCR, 2 μL of extracted DNA was added to GoTaq Probe qPCR master mix (Promega) with 0.8 μM primer and 0.4 μM probe. The cycling parameters were 95°C for 2 min, followed by 40 cycles of 95°C for 15 s and 60°C for 1 min, and the reactions were carried out in a QuantStudio Flex 6 (Applied Biosystems). The fluorescence signal was detected at the end of the extension phase of each cycle.

### 2.3. Histomorphometry analysis

For the histomorphometric analysis, the brain, liver, lung, and heart tissues were analyzed separately. The tissue area occupied by perivascular inflammatory cell aggregates (%) was calculated using the following equation: area occupied by perivascular immune cell aggregates (%) = total area occupied by inflammatory cell aggregates (μm^2^)/total tissue area (μm^2^) × 100. Histomorphometry was performed using ImageJ analysis software (11).

### 2.4. Development of an anti-PCV3 immunohistochemistry method

#### 2.4.1. Rabbits

Two 4-month-old New Zealand rabbits (each weighing 2 kg) were used for serum production. This research was conducted in accordance with the Norms for Animal Experimentation Ethics Committee of Universidade Federal do Rio Grande do Sul (process number 41581).

#### 2.4.2. *In silico* characterization of capsid protein for peptide selection

The genome of PCV3 strain 29,160 (GenBank accession number KT869077) was analyzed to identify the capsid protein ORF. Various bioinformatic methods were used to identify putative suitable antigenic peptide sequences in the deduced amino acid sequence of PCV-3 capsid protein (GenBank accession number ANO40512) that could be useful for generating antibodies. B-cell linear epitopes were identified using BepiPred 2.0 (12). Predictions were performed using an epitope threshold of 0.5, and antigenic epitope sequences of at least six amino with predicted epitope scores were noted. Regions with adequate antigenic indices were predicted using the Jameson-Wolf algorithm (13) in the LASERGENE software package (version 7.0.0). Regions of similarity among the capsid proteins of 100 different PCV3 isolates were determined by multisequence alignment using Clustal-X (14) in BioEdit version 7.2.5 (15). NetSurfP-3.0 was used to predict surface accessibility (16) and estimate antibody accessibility. Ultimately, two peptides were selected to produce anti-capsid protein antibodies. Peptide selection was based on the prediction of a high antigenic index and conservation among isolates. A template created with the Swiss-Model server (17) under default parameters was used to visualize the positions of the selected peptides in the protein and assembled virion.

#### 2.4.3. Production of anti-capsid protein sera

The peptides were synthesized by the Fmoc procedure (18) using an automated bench-top simultaneous multiple solid-phase peptide synthesizer (PSSM 8; Shimadzu). The peptides were deprotected with TFA and purified by semi-preparative HPLC using an Econosil C-18 column (10 μm, 22.5 × 250 mm) and a two-solvent system of trifluoroacetic acid (TFA)/H_2_O (1:1,000, solvent A) and TFA/acetonitrile (ACN)/H_2_O (1:900:100, solvent B). The sample was eluted at a flow rate of 5 mL/min with a gradient of 10–50% solvent B over 30 min or 30–60% solvent B over 45 min. A binary HPLC system from Shimadzu with an SPD-10AV Shimadzu UV–Vis detector coupled to an Ultrasphere C-18 column (5 μm, 4.6 × 150 mm) was used for analytical HPLC. The sample was eluted with a two-solvent system of H_3_PO_4_/H_2_O (1:1,000, solvent A) and ACN/H_2_O/H_3_PO_4_ (900:100:1, solvent B) at a flow rate of 1.0 mL/min and a 5–80% gradient of B over 10 min. The eluate was monitored by measuring the absorbance at 220 nm. The quality and purity of the peptides were checked using MALDI-TOF mass spectrometry (Bruker Daltons) and electrospray LC/MS-2020 (Shimadzu), which showed a single peak of the expected molecular weight.

The peptide was conjugated to keyhole limpet hemocyanin (KLH) or bovine serum albumin (BSA) as a carrier protein using glutaraldehyde (19). The carrier protein (5 mg/mL) and peptide (5 mg/mL) were resuspended in phosphate-buffered saline (PBS), and 0.2% glutaraldehyde was added to the protein/peptide solution and incubated with agitation for 1 h at room temperature. Glycine (1 M) was added to stop the reaction, and the complex was dialyzed overnight in PBS. The conjugate was composed of 1 mg peptide and 1 mg carrier/mL.

The peptide-carrier protein conjugates were mixed (1:1) with Montanide adjuvant (ISA 61 VG, Seppic). Each rabbit was subcutaneously inoculated with 500 μg of each peptide conjugate three times at 10-day intervals. Blood was collected 15 days after the last inoculation, and serum was obtained by centrifugation at 12,000 × *g* for 10 min at 4°C. The serum was stored at −20°C until use. The specificity and titer of the serum were determined against the BSA/peptide conjugate using a dot blot assay and ELISA. Briefly, 1 μg of conjugated peptide-BSA was spotted on a nitrocellulose membrane and dried at room temperature. The membrane was blocked with 5% skim milk in PBS for 1 h and then incubated with diluted serum overnight at 4°C. The membrane was then washed and incubated with alkaline phosphatase-conjugated anti-rabbit IgG (1:5,000) for 1 h at room temperature and detected using BCIP/TNBT chromogen.

#### 2.4.4. ELISA

The BSA-peptide conjugate (diluted in 0.02 M carbonate buffer, pH 9.6) was added to the wells of microtitration plates (200 ng per well) and incubated overnight at 4°C. The plates were washed three times and incubated for 1 h at 37°C with 5% non-fat dry milk in PBS to block non-specific binding and then incubated with serum (100 μL in 5% non-fat dry milk-PBS) for 1 h at 37°C. The plates were washed three times with 5% non-fat dry milk-PBS, and then incubated with peroxidase-conjugated anti-rabbit IgG for 1 h at 37°C. After three washes with PBS, the chromogen was added (3.4 mg σ-phenylenediamine, 5 μL 30% H_2_O_2_ in 0.1 M citrate–phosphate buffer, pH 5.0), and the plate was incubated for 5 min at room temperature. The reaction was stopped by adding 12.5% H_2_SO_4_, and the optical density (OD) was measured at 492 nm.

#### 2.4.5. Immunohistochemistry

FFPE tissues (brain, lung, heart, liver, spleen, vascular mesenteric plexus, and mesenteric lymph nodes) of a PCV3-positive piglet (case #5, confirmed by ISH, see Table 1) were cut into 3 μm thick sections and placed on positively charged, silanized slides. The tissue slides were heated at 60°C, immersed and deparaffinized in xylene, and hydrated in a graded series of ethanol (100–70%) (20). For all the tested protocols, endogenous peroxidase was blocked with 3% hydrogen peroxidase in methanol for 15 min, and then non-specific antibody binding was blocked for 7 min with 0.4% casein blocker diluted in PBS (Leica Biosystems, United Kingdom).

**Table 1:** Individual data about microscopic findings, ISH and IHQ results, of 17 piglets with PCV3-associated disease.

Cases	Tissues ISH +	Tissues IHQ +	Microscopic findings	
			Perivascular inflammatory infiltrate	Other findings
1	Heart, liver, lung, kidney, lymph node, skeletal muscle and adrenal gland.	Kidney, heart and mesenteric plexus.	Mesenteric plexus* and liver (M2); spleen* (M3); lung, lymph node, skeletal muscle, esophagus, SI, LI, heart, adrenal gland and kidney (M1).	Interstitial pneumonia (M1), Myocarditis (M2).
2	Spleen, lung, liver, heart, testicle and kidney.	Mesenteric plexus, heart, lung, kidney and spleen.	Mesenteric plexus*, pampiniform plexus*, spleen, lung, stomach (M2); heart, liver, kidney, adrenal gland, brain and skeletal muscle (M1).	Interstitial pneumonia (M2), Myocarditis (M2).
3	NP	N	Meseteric plexus*, pampiniform plexus (M3); liver, heart. Kidney, spleen (M2); LI (M1); CNS and lung (M1).	Interstitial pneumonia (M1), Myocarditis (M2), Gliosis (M1).
4	Kidney, lung and liver.	Mesenteric plexus.	Mesenteric plexus*, stomach, lung, kidney*, esophagus, heart, pancreas (M2); liver, brain (M1); SI and LI (F1).	Interstitial pneumonia (M1), Myocarditis (M2), Gliosis (F1).
5	Heart, CNS, kidney, adrenal gland, skeletal muscle, liver, stomach, skin, mesenteric plexus, mesenteric lymph node, tonsil and lung.	Lung, mesenteric plexus and heart.	Hearts, liver, kidney, lung, stomach (M2); skeletal muscle, mesenteric plexus, brain and LI (M1).	Interstitial pneumonia (D3), Myocarditis (M2+), Gliosis (M1).
6	Liver, lung, heart, CNS, spleen and tonsil.	Mesenteric plexus and heart.	Heart, spleen, kidney (M2); skeletal muscle, lung, mesenteric plexus, urinary bladder and CNS (M1).	Interstitial pneumonia (M1), Myocarditis, Miositis+ (M1), Gliosis (F1).
7	NP	Heart	Mesenteric plexus, pampiniform plexus, heart, CNS, kidney, skeletal muscle (M2); spleen, lung, liver, urinary bladder, brain and SI (M1).	Interstitial pneumonia (M1), Myocarditis (M2), Gliosis (M1).
8	NP	Heart and kidney.	Skeletal muscle, kidney, heart, spleen (M2); lymph node, mesenteric plexus, tonsil, CNS, liver and lung (M1); SI (F1).	Interstitial pneumonia (M1), Miositis (M1), Myocarditis (M1).
9	NP	N	Pampiniform plexus (M2); Mesenteric plexus, liver, spleen, lung, skeletal muscle and kidney (M1).	Interstitial pneumonia (M2), Myocarditis (M1).
10	NP	N	Heart* (M3); mesenteric plexus*, stomach, spleen, kidney* and lung (M2). SI, LI, urinary bladder, liver and brain (M1).	Myocarditis (M2+), Miositis (M1), Interstitial pneumonia (M3).
11	NP	Mesenteric plexus.	Spleen, heart (M2); skeletal muscle, mesenteric plexus, liver and kidney (M1).	Myocarditis (F1), Miositis (M1), Interstitial pneumonia (M1).
12	NP	Mesenteric plexus, heart and lung.	Mesenteric plexus*, urinary bladder, brain, skeletal muscle, spleen*, lung, kidney, heart (M2); liver and LI (M1).	Myocarditis (M1), Interstitial pneumonia (M2).
13	NP	Mesenteric plexus and heart.	Mesenteric plexus, pampiniform plexus, skeletal muscle, SI, spleen (M2); CNS, urinary bladder, heart, kidney and lung (M1).	Myocarditis (M1), Interstitial pneumonia (M1).
14	NP	Mesenteric plexus and heart.	Kidney, mesenteric plexus (M2); stomach, lung, brain, spleen, liver and heart (M1).	Myocarditis (M2), Interstitial pneumonia (M1), Gliosis (F2)
15	Skin, kidney, liver, lung, spleen, heart and mesenteric plexus.	Heart.	Mesenteric plexus* (M3); spleen, lung (M2); kidney, LI, esophagus, liver, brain, urinary bladder and heart (M1).	Gliosis (F1), Myocarditis (M2), Interstitial pneumonia (D3).
16	NP	Lung and heart.	Pampiniform plexus, spleen (M2); mesenteric plexus, brain, liver, kidney, skeletal muscle, heart and lung (M1).	Interstitial pneumonia (D3), Myocarditis (M1).
17	NP	Lung and heart.	Mesenteric plexus, pampiniform plexus, kidney, skeletal muscle, spleen, lung (M2); brain, heart and liver (M1).	Myocarditis (M2), Interstitial pneumonia (M1).

NP, Not Performed; N, Negative; SI, small intestine; LI, large intestine; F; focal; M, multifocal; D, Diffuse; 1, Mild; 2, Moderate; 3, Marked. *Vasculitis. +fibrosis.

Several parameters were varied to determine the optimal IHC protocol. In the first round, five different protocols for antigen retrieval were tested. The slides were treated with Protease XIV for 5 min (Protocol A), 10 min (Protocol B), or 15 min (Protocol C), or Proteinase K for 1 min (Protocol D), or 30 s (Protocol E). The tissues were then incubated with serum raised against capsid protein peptide (IgG-PCV3-1 or IgG-PCV3-2) or negative control for 2 h at 37°C in a dark chamber. Sera were diluted 1:50 in antibody diluent (0.87% NaCl, buffered with 10 mM Tris, pH 7.2, 15 mmol/L NaN_3_, and 0.1% BSA; Dako, Singapore). The tissues were incubated with biotinylated universal antibody (Dako) for 20 min at room temperature and with a streptavidin-peroxidase (Dako) for 20 min at room temperature.

In the second round, similar protocols were used, except epitope retrieval was conducted using a heat-induced epitope retrieval (HIER) method with 82.5 mM sodium citrate dihydrate buffer plus 17.5 mM citric acid (pH 6) in a digital pressure cooker at 96°C for 40 min (Protocol A), 125°C for 3 min (Protocol B), or 100°C for 10 min (Protocol C). Two additional protocols were included, in which the slides were incubated in a microwave oven at maximum power (10 w) for two or three cycles of 5 min each (Protocol D and Protocol E, respectively).

In round 3, all the steps were the same as described in round 2, except that Tris-EDTA buffer (10 mM Tris base, 1 mM EDTA, pH 9) was used for antigenic retrieval.

Four protocols were used in the fourth round. In protocols A and B, antigenic retrieval was performed using Tris-EDTA buffer (pH 9) and citrate buffer (pH 6), respectively, in a pressure cooker at 100°C for 40 min. In protocols C and D, antigenic retrieval was performed using Tris-EDTA buffer (pH 9) and citrate buffer (pH 6) in a pressure cooker at 125°C for 5 min. The detection systems for protocols A, B, C, and D were the same as those used in the previous rounds. However, simultaneously, four nearly identical protocols were performed, except that detection was conducted with the Universal HRP-Polymer kit, incubated with Post Primary reagent (mouse IgG anti-rabbit IgG; Leica Biosystems, United Kingdom) for 20 min at 37°C and incubated with Polymer reagent (Leica Biosystems) for 20 min at 37°C.

In the fifth round, different dilutions of IgG-PCV3-1 were tested, 1:50, 1:100, 1:200, and 1:300. Four protocols were performed, with the same procedure: peroxidase blocking for 15 min, antigenic retrieval with Tris-EDTA (pH 9) at 100°C for 40 min, and non-specific reaction blocking with 0.4% casein in PBS for 7 min. The tissue slides were incubated with IgG-PCV3-1 serum overnight at room temperature in a dark chamber. Then, the antigen–antibody detection system used was the Universal HRP-Polymer kit (Leica Biosystems).

In all protocols, antigen–antibody complexes were detected using the Romulin AEC chromogen kit (Biocare Medical, United States) and counterstained with Harris hematoxylin. The slides were dehydrated by immersion in a graded series of ethanol (70, 80, 90, and 100%), incubated in xylol for 10 min, and permanently mounted with Entellan® (Merck, Germany) and coverslipped. The detailed description of the tested IHQ protocols are in Supplementary Table 1.

Once the final protocol was established, all the 17 selected cases were subjected to PCV-3 IHC analysis of various tissues, such as the brain, lung, heart, liver, kidney, spleen, mesenteric vascular plexus, and mesenteric lymph nodes. Immunolabeling was evaluated based on the percentage of immunopositive cells and staining intensity, this was manually evaluated in each tissue and scored according to the methodology used by Kim et al. (21).

### 2.5. Statistical analyses

Comparison of the cycle threshold (Ct) in PCV3 qPCR among the different organs (brain, lymphoid tissues, lung, liver, and heart) in all cases was performed using the K-sample median test. Similarly, the areas occupied by perivascular aggregates of immune cells (histomorphometry) were compared to the Ct for each organ (brain, lung, liver, and heart) using the same test. Lymphoid tissue was not included in the comparison, since it consisted of different organs (the spleen and lymph nodes). If the results were significant, Tukey’s *post-hoc* test was performed. The Spearman correlation coefficient (Rs) between the Ct values for PCV3 qPCR and the areas occupied by perivascular immune cell aggregates (histomorphometry) was calculated for each organ. All statistical analyses were performed using commercial software (SPSS Statistics for Windows, Version 22.0; IBM, Armonk, NY, United States). Statistical significance was set at *p* < 0.05.

## 3. Results

### 3.1. Gross and microscopic observations

**Figure 1:**
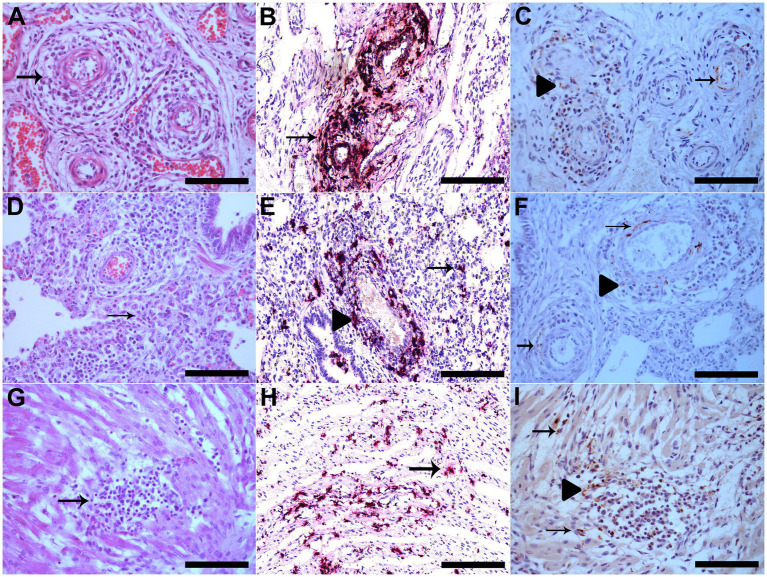
**(A)** The vessel walls of the mesenteric plexus are disrupted by marked infiltration of lymphocytes, histiocytes and plasma cells, besides fibrinoid degeneration (arrow), HE, Bar. 120 μm. **(B)** Vascular mesenteric plexus with PCV3 staining in inflammatory cells and vessel walls which is shown in red (arrow), hematoxylin was used as a counterstain to dye the tissue section for ISH Bar. 120 μm. **(C)** Vascular mesenteric plexus with PCV3 immunolabeling in the cytoplasm of inflammatory cells in perivascular areas (arrowhead) and in cytoplasm and nucleus of cells of smooth muscle of arteries (arrow), IHQ, AEC, Bar. 120 μm. **(D)** Lung with alveolar septa diffusely distended by lymphocytes and macrophages (interstitial pneumonia) (arrow), HE, Bar. 120 μm. **(E)** Lung with multifocal PCV3 staining in inflammatory cells in interstitial (arrow) and in perivascular areas, which is shown in red (arrowhead), ISH, Bar. 120 μm. **(F)** Lung with PCV3 immunolabeling in the cytoplasm of inflammatory cells in perivascular areas (arrowhead) and in cytoplasm and nucleus of cells of smooth muscle of arteries (arrow), IHQ, AEC, Bar. 120 μm. (**G)** area of mild lymphohistiocytic myocarditis (arrow), HE, Bar. 120 μm. **(H)** Cardiomyocytes with multifocal PCV3 staining, which is shown in red (arrow), ISH, Bar. 120 μm. **(I)** Heart with PCV3 immunolabeling in the cytoplasm of inflammatory cells (arrowhead) and in cytoplasm of cardiomyocytes (arrow), IHQ, AEC, Bar. 120 μm.

At post-mortem examination, all piglets with PCV3-associated disease had similar gross findings. The lungs did not collapse because of the marked interlobular edema. Microscopically, the affected piglets had multisystemic lymphoplasmacytic and histiocytic perivasculitis (Figure 1A) and lymphohistiocytic interstitial pneumonia (Figure 1D). Multifocal areas of mild lymphohistiocytic myocarditis (Figure 1G), myositis, and gliosis were also observed. The descriptions of individual microscopic lesions are presented in Table 1. ISH staining was observed in the inflammatory cells in perivascular multisystemic areas and in the smooth muscle of arteries (Figure 1B) and in the interstitial lymphohistiocytic infiltrates in the lungs (Figure 1E), cardiomyocytes (Figure 1H), and neurons of the brain [see (2) for details].

### 3.2. qPCR results

**Figure 2:**
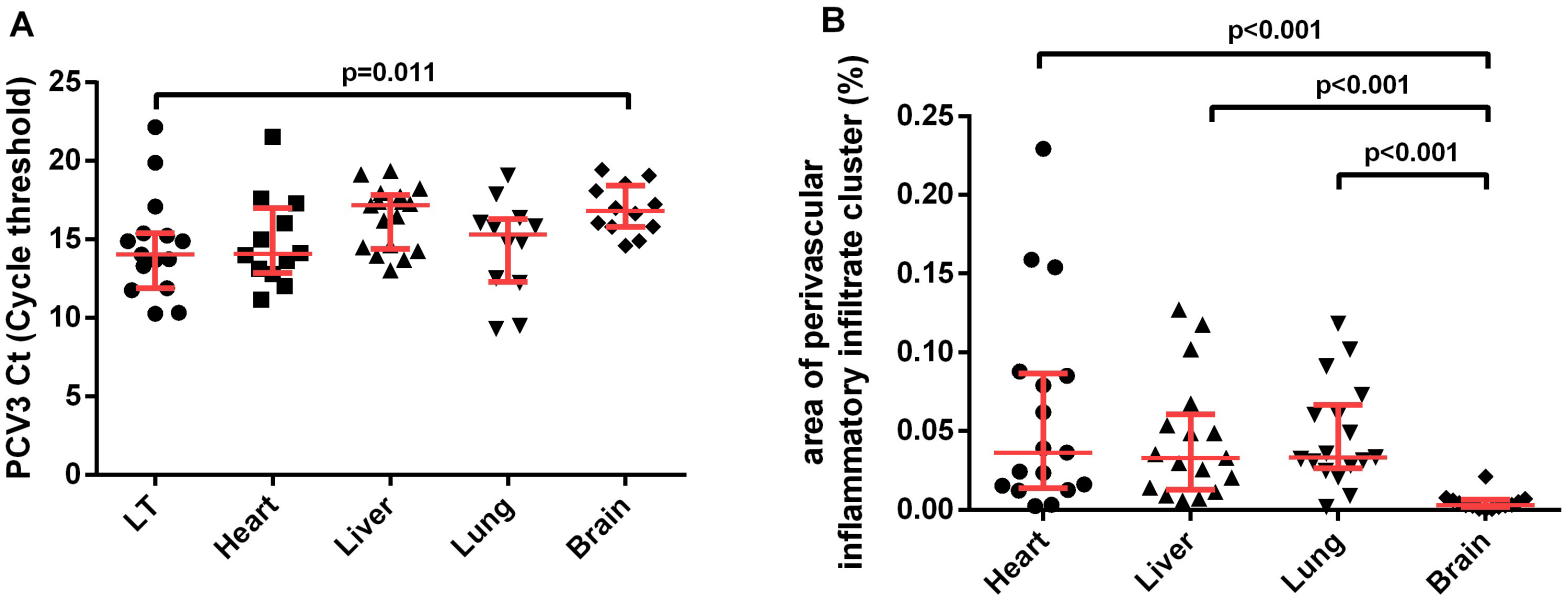
Pairwise comparison of variables **(A)** PCV3 Ct (Cycle threshold) value and **(B)** area of perivascular inflammatory infiltrate cluster (%). Interquartile ranges and medians were used to summarize the data and *p-*values from the Tukey test. K-sample median test results of PCV3 Ct value (*p* < 0.009) and area of perivascular inflammatory infiltrate cluster (*p* < 0.001) not showed.

A significant difference was observed in the median qPCR Ct among the analyzed tissues (K-sample median test, *p* = 0.009). In the pairwise comparison, lymphoid tissue had a lower qPCR Ct value than the brain (Tukey test, *p* = 0.011; Figure 2A). The median area occupied by immune cell aggregates also differed between organs (K-sample median test, *p* < 0.001). The pairwise comparison showed a smaller area in the brain than in the liver, heart, and lung (Tukey test, *p* < 0.001; Figure 2B). There was no correlation between the PCV3 qPCR Ct value and the tissue area occupied by perivascular immune cell aggregates in the heart (Rs = 0.357; *p* = 0.255), liver (Rs = −0.044; *p* = 0.866), lungs (Rs = −0.252; *p* = 0.430), or brain (Rs = −0.277; *p* = 0.384) (data not shown).

### 3.3. PCV3 immunohistochemistry assay development

**Figure 3:**
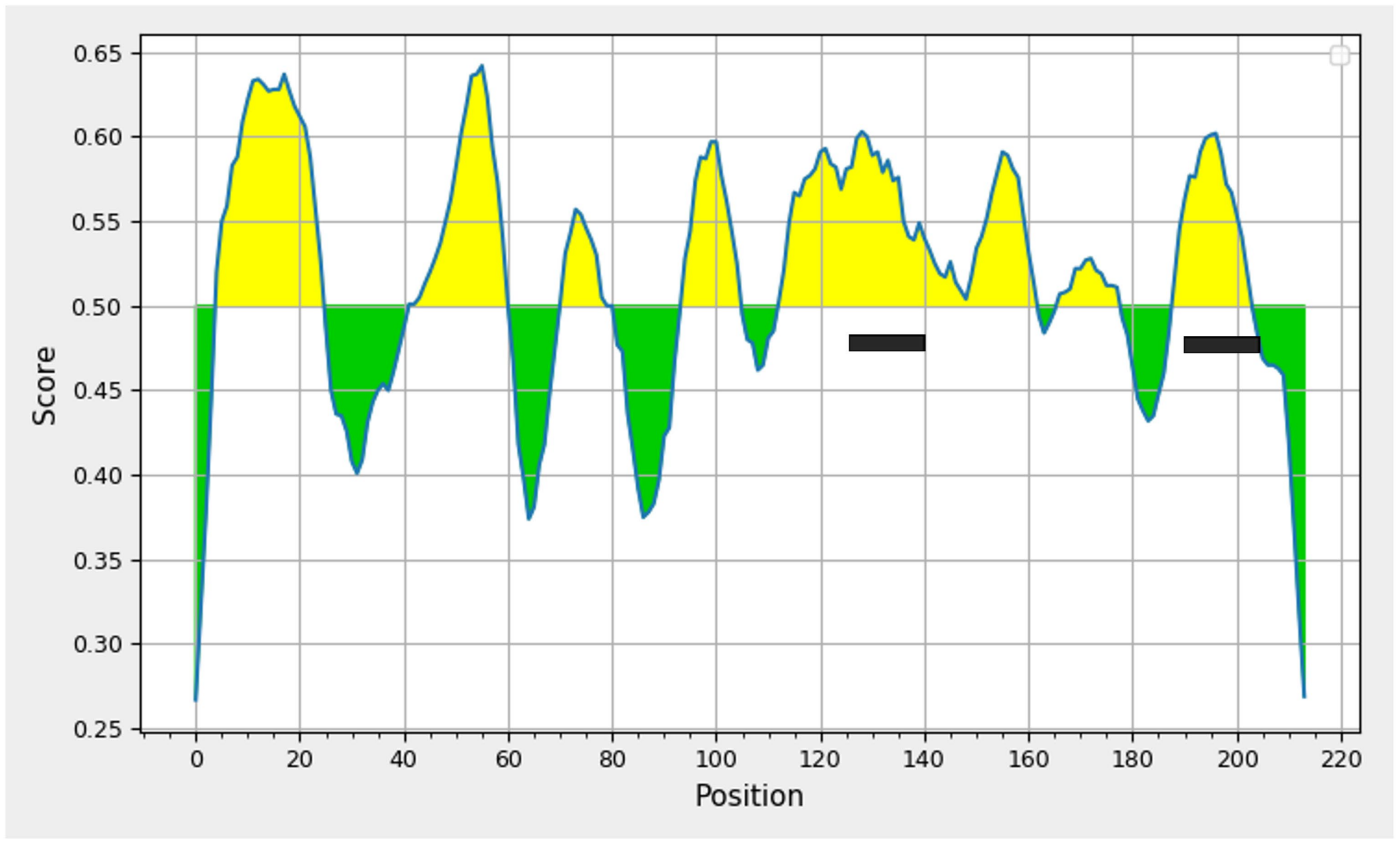
Prediction of linear B-cell epitopes deduced amino acid sequence of porcine circovirus type 3 capsid protein using Bepipred linear epitope prediction tool 2.0, threshold value 0.5 Yellow areas above the threshold are predicted to be part of a B cell epitope. The two peptides selected are highlighted.

**Figure 4:**
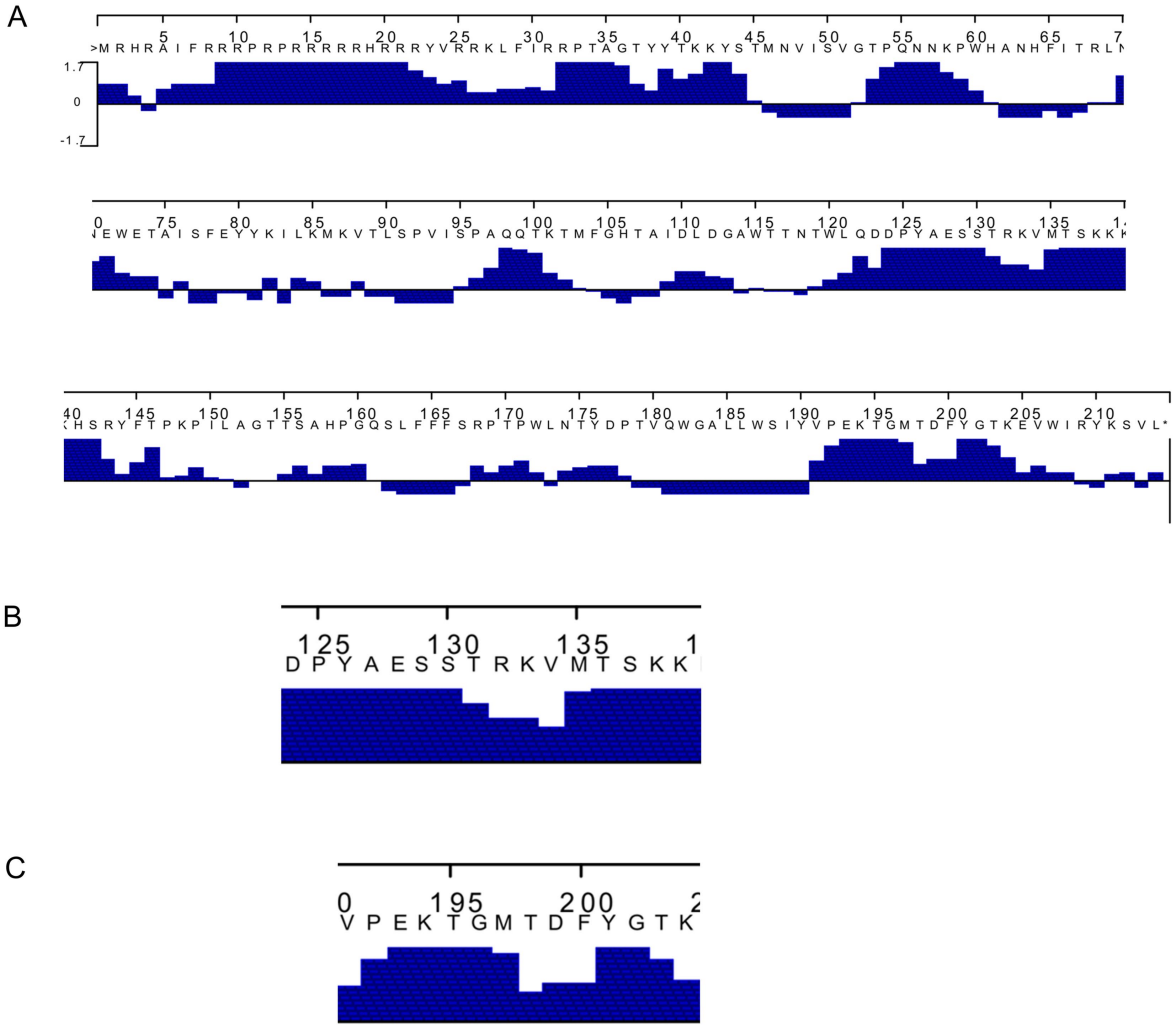
**(A)** Prediction of the antigenic index of deduced amino acid sequence of Porcine circovirus type 3 capsid protein by Jameson–Wolf algorithm highlighting **(B,C)** the two peptides selected.

**Figure 5:**
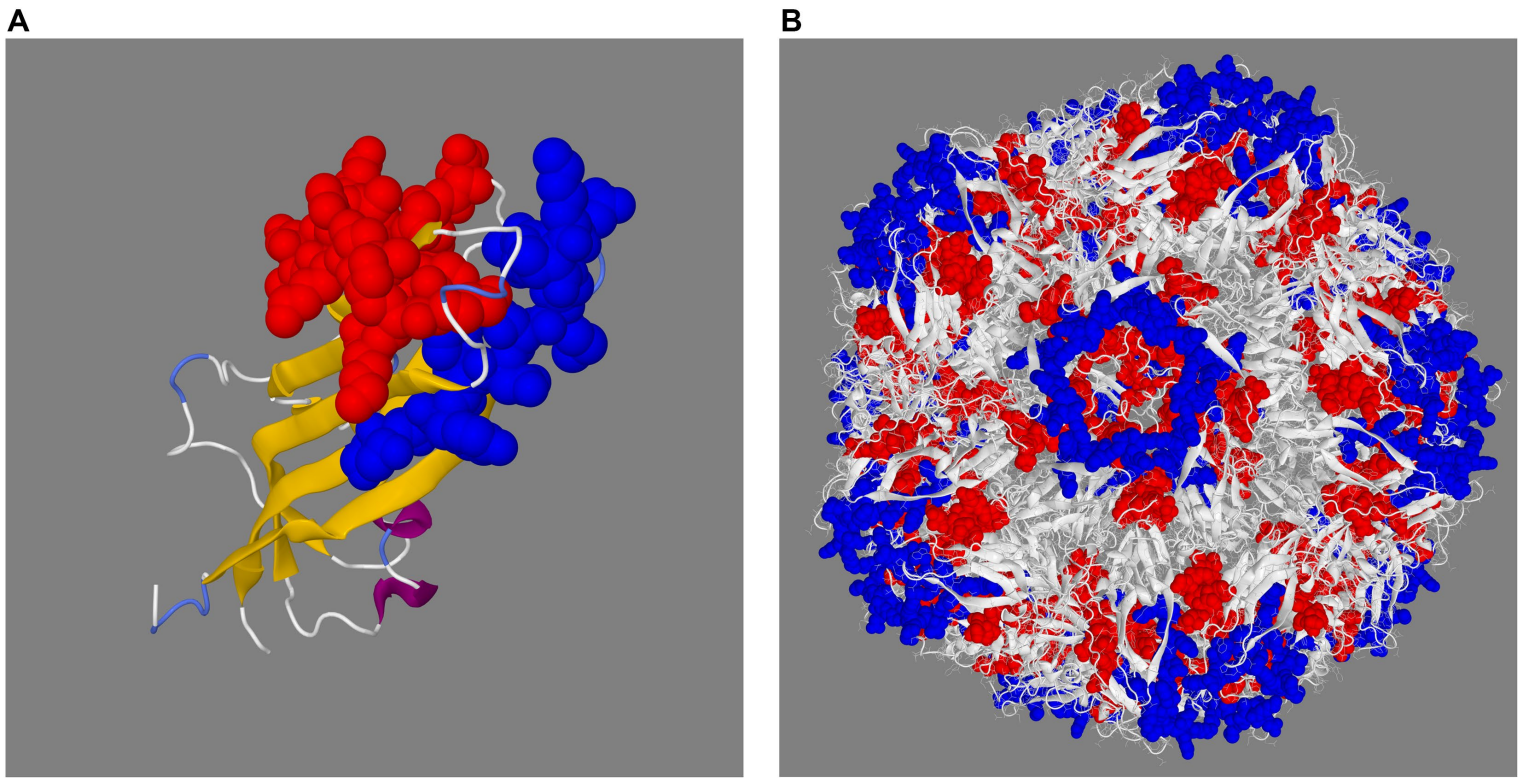
Prediction of the 3D structure of the capsid protein **(A)** and full capsid of porcine circovirus type 3 **(B)**. The 3D structure shows the two selected peptides in space filling representation in red (PCV3-1) and blue (PCV3-2).

The PCV3 capsid protein sequences of 100 PCV3 isolates from different regions of the world showed high conservation, with >98.6% percent identity at the amino acid level (Supplementary Datasheet 1). Linear epitopes of the capsid protein were predicted using the BepiPred algorithm, and the best linear epitopes are shown in Figure 3, which are likely the most immunogenic peptides of the capsid protein. Analysis of surface accessibility predicted that the amino acid residues included in the synthetic peptides may be exposed in the capsid protein (Supplementary Figure 1). Jameson-Wolf analyses also indicated the presence of antigenic regions in the capsid protein (Figure 4) that overlapped with the predicted B-cell epitopes, suggesting potential regions for peptide synthesis. As a complementary analysis of the PCV3 capsid, we identified the best templates in the PDB protein databank: the capsids for 6RPO bat circovirus (identity: 32%, coverage: 81%) and porcine circovirus 2 (identity: 32%, coverage: 84%); template coverage was high, but identity was low. The Ramachandran plot of the modeled PCV3 protein showed 94.73% residues in the favored region, 3.98% in the allowed regions, and only 1.28% of the residues were in the outlier region, indicating the suitability of the deduced structure (Figure 5 and Supplementary Figure 2). This experimental approach enabled the selection of two capsid protein peptides to produce anti-capsid protein antibodies: PCV3-1 (DPYAESSTRKVMTSKK) and PCV3-2 (VPEKTGMTDFYGTK). Peptide selection was based on the prediction of higher antigenic/antigenic indices and conservation among virus isolates.

**Figure 6:**
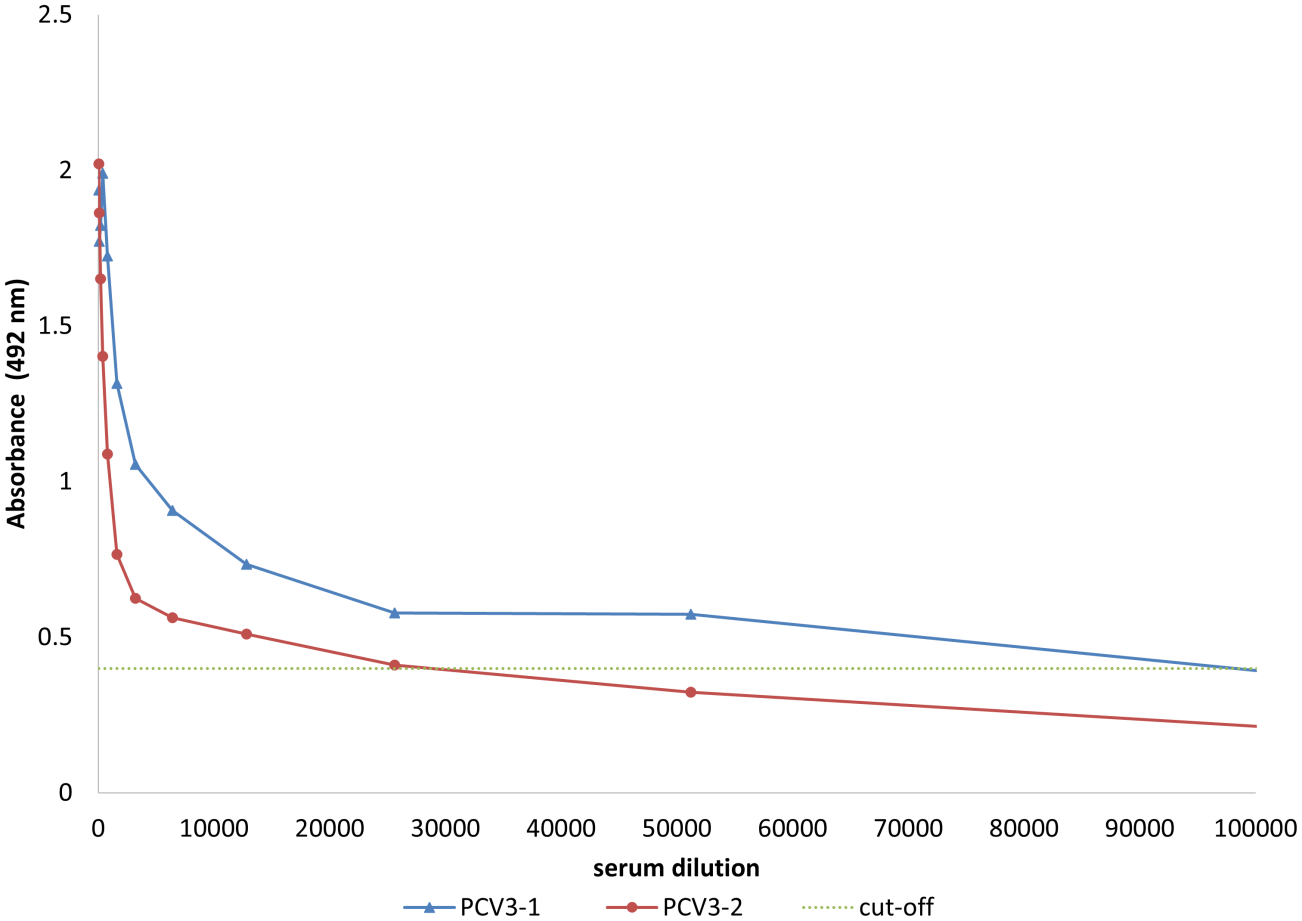
ELISA evaluation of serum from rabbits inoculated with synthetic peptides derived from capsid protein conjugated to KLH. The rabbit sera (anti-PCV3-1 peptide and anti-PCV3-2 peptide) specifically recognize synthetic peptides conjugated to BSA. Results corresponding to diluted serum samples are presented. Cut-off is shown by the dotted line.

Since short peptides are not highly immunogenic, PCV3-1 and PCV3-2 peptides were covalently conjugated to KLH protein for rabbit immunization. BSA was used as a carrier to improve coating of the ELISA plate during serum analysis. Two rabbits were independently immunized with synthetic polypeptides PCV3-1 and PCV3-2. The peptide antisera recognized the corresponding peptide-BSA conjugates with titers >25,000 (Figure 6), indicating the immunogenicity of the synthesized peptides. These antisera were used to develop an IHC assay to detect PCV3 in paraffin-embedded piglet tissues.

All IHC slides were evaluated using an optical microscope under 200× and 400× magnifications. The tissue slides from the first round of test IHC protocol testing showed no antigen labeling. In the second round, the sections incubated with IgG-PCV3-1 sera showed rare cytoplasmic granular labeling in cardiomyocytes (1% weakly positive cells), while the remaining protocols resulted in no antigen labeling.

In the third round of protocols, the sections incubated with IgG-PCV3-1 serum had mild cytoplasmic granular labeling (5% positive cells) in cardiomyocytes, inflammatory cells in the myocardium and in pulmonary interstitial spaces, and inflammatory cells surrounding and disrupting vessel walls. The remaining protocols had no evidence of labeling.

In the fourth round, the slides subjected to antigen recovery using Tris-EDTA (pH 9) at 100°C for 40 min and incubated with IgG-PCV3-1 serum (A and E) had cytoplasmic granular labeling (20% moderately positive cells). These cells were composed of cardiomyocytes, inflammatory cells in the myocardium and pulmonary interstitial spaces, and inflammatory cells surrounding and disrupting the vessel walls. In addition, when streptavidin-biotin was used as the detection system, only 5% labeling was observed, whereas when polymer (Leica) was used, detection increased to 20%.

The slides in the fifth round were incubated with a 1:200 dilution of IgG-PCV3-1 serum, which resulted in strong immunolabeling of 30% of target cells. The immunolabeling was multifocal and granular, mostly in the cytoplasm but also in the nucleus. Immunolabeling was observed mainly in the muscular layer of the vascular wall, cardiomyocytes, and histiocytes in the areas of myocarditis and the alveolar septa. The positive results for individual piglets are shown in Table 1. The negative controls showed no viral protein staining in the pig tissues.

In the 17 selected cases, immunolabeling was observed in various tissues, such as vascular mesenteric plexus (9/17) (Figure 1C), lungs (4/17) (Figure 1F), the heart (12/17) (Figure 1I), kidneys (3/17), and spleen (1/17). No immunolabeling was observed in the brain, mesenteric lymph nodes, or liver. The individual data are presented in Table 1.

## 4. Discussion

The importance of PCV3 infection, a pig disease with varied symptoms, in the swine industry has been increasing, as it has spread worldwide (22, 23). Currently, ISH is the standard technique for detecting PCV3 infection (24). However, this method requires expensive facilities, special equipment, and well-trained technicians. The present study was conducted to evaluate the relationship between PCV3 viral load and histopathological findings in perinatal piglet tissues and to develop an IHC technique for virus detection in lesions.

Perivascular lymphoplasmacytic and histiocytic infiltrates, occasionally occurring in association with vasculitis, was a constant finding in several organs of all analyzed cases, similar to what has been previously described for PCV3 infection in neonatal piglets (3). Another common microscopic finding in all piglets in the present study was myocarditis, characterized by necrosis and fragmentation of cardiomyocytes, with multifocal areas of lymphohistiocytic infiltration. These findings are similar to those previously described for piglets naturally infected with PCV3 (3, 7, 24). Interstitial pneumonia was also a constant finding, of varying intensity, with alveolar septa distended by lymphohistiocytic infiltrates and prominent lymphoplasmacytic and histiocytic perivascular infiltrates in most cases, as was previously described in piglets (24) and aborted fetuses (4). Myocarditis, cardiac vasculitis, perivasculitis, and interstitial pneumonia were previously statistically associated with PCV3-infected pigs with low qPCR Ct values (25). In skeletal muscle, we observed prominent perivascular non-suppurative infiltrates (12/17) and myositis (5/17), similar to what has been described in aborted piglets caused by PCV3 infection (4). Gliosis was observed in 5 of 17 piglets, consistent with features previously found in perinatal piglets (3) and aborted fetuses (4). The prominent and constant microscopic lesions in tissues, including the mesenteric vascular plexus, heart, lung, CNS, and skeletal muscle, highlights the importance of collecting these organs for adequate diagnosis of PCV3-associated disease.

Virus titer and tissue tropism have been proposed as factors determining PCV3 virulence (26). In our analysis, qPCR Ct values were lower in lymphoid organs (spleen and mesenteric lymph nodes) than in the CNS, indicating a higher viral load, whereas no significant difference was observed between lymphoid organs and other organs, such as the lungs, heart, and liver. Although variation in Ct values has been well described in laboratory infection, varying according to the day post-inoculation (26), in natural infections, as in our study, the Ct values were similar to those previously described by Deim et al. (27), ranging from 28 to 20. However, we detected PCV3 in all analyzed organs, which differs from that in aborted fetuses and stillborn piglets (27), in which PCV3 detection varied considerably between organs and was most frequently detected in lymphoid organs. We hypothesize that this information points to a lack of an effect in the sampled organs, as all samples were positive by qPCR. The virus can multiply in endothelial cells, cardiomyocytes, and inflammatory cells (26), and this has been demonstrated by ISH (3–5). Virus levels did not vary among samples of different tissues, as PCV3 invades multiple tissues and organs. Although the lymphoid organs had lower qPCR Ct values than CNS tissue, qPCR was performed on pooled tissues (spleen and lymph nodes); therefore, it was not possible to compare our results to previous findings (27).

The lack of a correlation between the area of perivascular inflammatory infiltrates and the qPCR Ct values in the evaluated tissues suggested that the inflammatory lesions are not associated with viral load. The perivascular area occupied by inflammatory infiltrates in the brain was smaller than that in other organs. Whether this low inflammation in the CNS is a consequence of immunoregulatory mechanisms that reduce excessive inflammatory responses in immune-privileged tissues (28) or it is related to the pathogenesis of the virus remains unknown. This mirrors previous studies in which CNS findings did not appear in all newborn piglets nor was observed in 11 weaned piglets (3). Despite the reduced area of perivascular inflammatory infiltrates and discrete gliosis in the CNS tissue and qPCR Ct values similar to those in other evaluated tissues, it is compatible with the pathogenesis of the virus, which not only multiplies within immune cells but also in vessel walls (26). Despite the small sample size, this study yielded valuable insights. To enhance the ability to identify significant differences, future research can further improve the sensibility and specificity of the tests.

Through IHC, specific proteins can be detected in cells and tissues due to antibody–antigen binding (20), whereas in ISH, specific DNA and RNA sequences are detected in cells and tissues by hybridizing a probe to a target sequence (29). All ISH-positive cases were positive in IHC for at least one organ. PCV3 staining by IHC was granular, predominantly in the cytoplasm of inflammatory cells, vascular walls, and cardiomyocytes. A comparative visual analysis of tested tissues showed that IHC labeling was lower than ISH labeling.

In the present study, EDTA was superior to citrate buffer during epitope retrieval. This result is in accordance with previous observations. Although the mechanism involved in heat-induced epitope retrieval (HIER) is unknown, it has been hypothesized that tissue-bound calcium ions might mask some antigens during fixation; therefore, the use of an ion chelator, such as EDTA, can be more effective than citrate buffer (30, 31). Also, lower efficiency has been observed using protease-induced epitope retrieval (PIER) in other studies, since PIER can lead to alterations in cell morphology and destruction of epitopes; therefore, PIER is recommended for detecting a low number of antigens (32).

In a previous study, heating at a high temperature of 100°C for a short time (e.g., 10 min) yielded better results than heating at a low temperature for a longer time (33). We observed similar results when we used a lower temperature for a long time, as the results were not adequate; however, when we used a high temperature (100°C) for 40 min, we obtained adequate results.

The detection systems used for amplification of the target include the labeled streptavidin biotin, avidin biotin complex, phosphatase anti-phosphatase, polymer-based detection, and tyramine amplification methods. However, when compared to standard IHC methods, polymeric and tyramine-based amplification methods have at least 50-fold greater sensitivity (34). We observed a similar finding in the fourth round of protocol testing, in which the amount of labeling was increased when we used a polymer-based detection system instead of the streptavidin-biotin method.

Despite careful epitope selection, the serum against peptide PCV3-2 did not detect protein in the IHC experiments. This is a common problem when serum is raised against a peptide, since synthetic peptide sequences sometimes lack the three-dimensional structure of the native protein. Another reason for the serum failure is the presence of post-translational modifications in the protein spanning the selected peptide region or an association with another protein, which can alter epitope recognition and binding (35, 36).

Our study revealed that 3 out of 17 cases did not show immunolabeling. Further investigation is needed to determine whether this is due to limitations in the sensitivity of the IHC technique or unidentified characteristics of the cases.

IHC has continually evolved, and several protocols have been developed to identify antigens in tissues. Nonetheless, its sensitivity and specificity are dependent on the conditions and reagents used, and detection can be affected by inadequate or unstandardized conditions (21, 37, 38).

Freezing prior to tissue fixation is not recommended because freezing can rupture cell membranes, causing proteins leak out of the cells, which can interfere with immunolabeling (39). It can also cause morphological changes (40). Nevertheless, samples from two cases (piglets 11 and 13) in this study were frozen before fixation, and immunostaining was detected in some tissues (vascular mesenteric plexus and heart).

The detection of viral load, microscopic lesions, or antigen by IHC can vary greatly depending on the location and time of tissue sampling, since viral replication requires a variable period of time from adsorption to virus release (41). Furthermore, recirculation of infected immune cells, such as lymphocytes, could present viral proteins, but may not be detectable by IHC due to their scarce cytoplasm and the absence of cluster formation.

Variations in the time before tissue processing and fixation can also be a source of variability in IHC. Proper tissue fixation with buffered formalin is recommended to prevent autolysis and preserve cell morphology. Furthermore, overfixation can cause irreversible damage to some epitopes; therefore, 24 h of fixation is recommended (42, 43). In our study, we did not use buffered formaldehyde, and the tissues remained fixed for more than 24 h (48–72 h). Thus, we hypothesized that this could have interfered with the IHC results.

The present study is the first to provide a comprehensive description of the development of an IHC method for the detection of PCV3-associated disease in swine. Although other IHC methods for detection of PCV3 have been described by Palinski et al. (44) and Jiang et al. (45), these authors did not publish a detailed description of the method nor did they demonstrate consistent data or immunostaining. This IHC technique has been proven to be a useful and reliable method for detecting PCV3. In cases where further diagnostic confirmation is needed, PCR can be performed.

## 4. Limitation

The limitation of our study is that we were not able to perform ISH in all samples and neither compare the ISH results with IHC results. The interpretations were based more on qualitative results than quantitative results. The present data should be useful for improving routine diagnostics of PCV3-associated disease in swine. However, it is important to highlight that quantitative results are easier to replicate, whereas qualitative results are more subjective to interpretation. Future studies comparing a larger number of cases using PCR, IHC and ISH are valuable to improve the importance of the findings of this study.

## 5. Conclusion

The most frequent microscopic lesions detected in our cases of PCV3 infection were multisystemic non-suppurative lymphoplasmacytic and histiocytic periarteritis; one of the most affected tissues was the mesenteric vascular plexus, which is often associated with vasculitis. Tissues such as the heart, lung, CNS, and skeletal muscle also had prominent microscopic lesions. This demonstrates the importance of collecting these tissues for the presumptive histological diagnosis of PCV3.

Comparison of the qPCR Ct values for different tissues (CNS, lung, heart, liver, spleen, and lymph nodes) showed no significant differences, except between lymphoid organs (spleen and lymph node) and CNS tissue, in which the lymphoid organs had a higher viral load. There was no correlation between the qPCR Ct values and the area of perivascular inflammatory infiltrates in different tissues.

The developed anti-PCV3 IHC method recognized the target protein with granular immunolabeling, mainly in the cytoplasm of the tested tissues. In conclusion, IHC allowed the identification of an association between the presence of the infectious agent within microscopic tissue lesions, which will be useful for future investigations of PCV3 infection, diagnosis, pathogenesis, and epidemiology.

## Data availability statement

The datasets presented in this study can be found in online repositories. The names of the repository/repositories and accession number(s) can be found in the article/Supplementary material.

## Ethics statement

Ethical review and approval was not required for the study of animals in accordance with the terms of Brazilian Federal Law No. 11,794/08. Written informed consent was obtained from the owners for the participation of their animals in this study.

## Author contributions

FM, IV, and DD conceived and designed the experiments. FM and IV performed the experiments. TN crucial contributed in performing immunohistochemistry tests. IV and DD contributed to reagents, materials, and analysis tools. FM, BA, IV, and DD drafted the article. FM, BA, BS, CP, LV, LB, GS, FV, TN, IV, and DD contributed to critical revision of the article. All authors contributed to the article and approved the submitted version.

## Funding

This work was supported by grants from Conselho Nacional de Desenvolvimento Científico e Tecnológico (CNPq) #159522/2019-6, 405763/2018-2, and #465678/2014-9 INCT-EM (Instituto Nacional de Ciência e Tecnologia de Entomologia Molecular) and Fundação de Amparo à Pesquisa do Estado do Rio Grande do Sul (FAPERGS)# 21/2551-0002221-3 (Brazil), Coordenação de Aperfeiçoamento de Pessoal de Nível Superior (CAPES)—Finance Code 001 and Pró-reitoria de Pesquisa da Universidade Federal do Rio Grande do Sul (Propesq/UFRGS).

## Conflict of interest

TW was employed by the company Antech Diagnostics.

The remaining authors declare that the research was conducted in the absence of any commercial or financial relationships that could be construed as a potential conflict of interest.

## Publisher’s note

All claims expressed in this article are solely those of the authors and do not necessarily represent those of their affiliated organizations, or those of the publisher, the editors and the reviewers. Any product that may be evaluated in this article, or claim that may be made by its manufacturer, is not guaranteed or endorsed by the publisher.
