# Corrigendum: Porcine circovirus type 3: immunohistochemical detection in lesions of naturally affected piglets

**DOI:** 10.3389/fvets.2023.1229149

**Published:** 2023-06-26

**Authors:** Franciéli Adriane Molossi, Bruno Albuquerque de Almeida, Bianca Santana de Cecco, Caroline Pissetti, Lauren Ventura, Luciano Brandalise, Gustavo Simão, Fabio Vanucci, Tatiane Terumi Negrao Watababe, Itabajara da Silva Vaz, David Driemeier

**Affiliations:** ^1^Faculdade de Veterinária, Universidade Federal do Rio Grande do Sul, Porto Alegre, Brazil; ^2^Department of Pathobiological Sciences, Louisiana State University, Baton Rouge, LA, United States; ^3^Centro de Diagnóstico de Sanidade Animal (CEDISA), Concórdia, Brazil; ^4^Agroceres Pic, Rio Claro, Brazil; ^5^Veterinary Diagnostic Laboratory, University of Minnesota, St. Paul, MN, United States; ^6^Department of Population Health and Pathobiology, College of Veterinary Medicine, North Carolina State University, Raleigh, NC, United States; ^7^Antech Diagnostics, West Olympic Blvd, Los Angeles, CA, United States; ^8^Centro de Biotecnologia, Universidade Federal do Rio Grande do Sul, Porto Alegre, Brazil; ^9^Instituto Nacional de Ciência e Tecnologia - Entomologia Molecular, Rio de Janeiro, Brazil

**Keywords:** diagnosis, pathology, PCV3, piglets, immunohistochemistry

In the published article, there was an error in **affiliation(s)** 6 and 7. Instead of “^6^ Department of Population Health and Pathobiology, College of Veterinary Medicine, North Carolina State University, Los Angeles, CA, United States.”

The affiliations should be corrected as follows:

^6^ Department of Population Health and Pathobiology, College of Veterinary Medicine, North Carolina State University, Raleigh, NC, United States

^7^ Antech Diagnostics, West Olympic Blvd, Los Angeles, CA, United States

In the published article, there was an error regarding the **affiliation(s)** of the author: Tatiane Terumi Negrao Watababe. As well as having affiliation(s) 6, she should also have “^7^ Antech Diagnostics, West Olympic Blvd, Los Angeles, CA, United States.”

In the published article, there was an error in the Conflict of Interest statement. The statement should have read “Author TN was employed by the company Antech Diagnostics. The remaining authors declare that the research was conducted in the absence of any commercial or financial relationships that could be construed as a potential conflict of interest.”

The authors apologize for this error and state that this does not change the scientific conclusions of the article in any way. The original article has been updated.

