# Supplementary material for: Porcine circovirus type 3: immunohistochemical detection in lesions of naturally affected piglets
Source: Front Vet Sci. 2023 May 4;10:1174718. doi: 10.3389/fvets.2023.1174718 (PMC10192697; doi:10.3389/fvets.2023.1174718)
Supplement: Supplementary file 1 [file Table_1.docx]

Supplementary Table 1: Detailed description about the tested IHQ protocols.

| **First round** | **Antigen retrieval** | **Serum dilution** | **Detection system** |
| --- | --- | --- | --- |
| **Protocol A** | Protease XIV for 5 min | 1:50 | biotinylated universal antibody (Dako), 20 min at room temperature; streptavidin-peroxidase (Dako) for 20 min at room temperature. |
| **Protocol B** | Protease XIV for 10 min | 1:50 | biotinylated universal antibody (Dako), 20 min at room temperature; streptavidin-peroxidase (Dako) for 20 min at room temperature. |
| **Protocol C** | Protease XIV for 15 min | 1:50 | biotinylated universal antibody (Dako), 20 min at room temperature; streptavidin-peroxidase (Dako) for 20 min at room temperature. |
| **Protocol D** | Proteinase K for 1 min | 1:50 | biotinylated universal antibody (Dako), 20 min at room temperature; streptavidin-peroxidase (Dako) for 20 min at room temperature. |
| **Protocol E** | Proteinase K for 30 sec | 1:50 | biotinylated universal antibody (Dako), 20 min at room temperature; streptavidin-peroxidase (Dako) for 20 min at room temperature. |
| **Second round** |  |  |  |
| **Protocol A** | Citric acid (pH 6) in a digital pressure cooker at 96°C for 40 min | 1:50 | biotinylated universal antibody (Dako), 20 min at room temperature; streptavidin-peroxidase (Dako) for 20 min at room temperature. |
| **Protocol B** | Citric acid (pH 6) digital pressure cooker 125°C for 3 min | 1:50 | biotinylated universal antibody (Dako), 20 min at room temperature; streptavidin-peroxidase (Dako) for 20 min at room temperature. |
| **Protocol C** | Citric acid (pH 6) digital pressure cooker  100°C for 10 min | 1:50 | biotinylated universal antibody (Dako), 20 min at room temperature; streptavidin-peroxidase (Dako) for 20 min at room temperature. |
| **Protocol D** | Citric acid (pH 6) Two cycles of 5 min each in a microwave oven maximum power (10 w) | 1:50 | biotinylated universal antibody (Dako), 20 min at room temperature; streptavidin-peroxidase (Dako) for 20 min at room temperature. |
| **Protocol E** | Citric acid (pH 6)  Three cycles of 5 min each in a microwave oven maximum power (10 w) | 1:50 | biotinylated universal antibody (Dako), 20 min at room temperature; streptavidin-peroxidase (Dako) for 20 min at room temperature. |
| **Third round** |  |  |  |
| **Protocol A** | Tris-EDTA buffer pH 9  in a digital pressure cooker at 96°C for 40 min | 1:50 | biotinylated universal antibody (Dako), 20 min at room temperature; streptavidin-peroxidase (Dako) for 20 min at room temperature. |
| **Protocol B** | Tris-EDTA buffer pH 9  digital pressure cooker 125°C for 3 min | 1:50 | biotinylated universal antibody (Dako), 20 min at room temperature; streptavidin-peroxidase (Dako) for 20 min at room temperature. |
| **Protocol C** | Tris-EDTA buffer pH 9  digital pressure cooker  100°C for 10 min | 1:50 | biotinylated universal antibody (Dako), 20 min at room temperature; streptavidin-peroxidase (Dako) for 20 min at room temperature. |
| **Protocol D** | Tris-EDTA buffer pH 9 Two cycles of 5 min each in a microwave oven maximum power (10 w) | 1:50 | biotinylated universal antibody (Dako), 20 min at room temperature; streptavidin-peroxidase (Dako) for 20 min at room temperature. |
| **Protocol E** | Tris-EDTA buffer pH 9 Three cycles of 5 min each in a microwave oven maximum power (10 w) | 1:50 | biotinylated universal antibody (Dako), 20 min at room temperature; streptavidin-peroxidase (Dako) for 20 min at room temperature. |
| **Fourth round** |  |  |  |
| **Protocol A** | Tris-EDTA buffer pH 9  digital pressure cooker  100°C for 40 min | 1:50 | biotinylated universal antibody (Dako), 20 min at room temperature; streptavidin-peroxidase (Dako) for 20 min at room temperature. |
| **Protocol A1** | Tris-EDTA buffer pH 9  digital pressure cooker  100°C for 40 min | 1:50 | Post Primary reagent (mouse IgG anti-rabbit IgG; Leica Biosystems, UK) for 20 min at 37°C and incubated with Polymer reagent (Leica Biosystems) for 20 min at 37°C. |
| **Protocol B** | citrate buffer (pH 6) digital pressure cooker  100°C for 40 min | 1:50 | biotinylated universal antibody (Dako), 20 min at room temperature; streptavidin-peroxidase (Dako) for 20 min at room temperature. |
| **Protocol B1** | citrate buffer (pH 6) digital pressure cooker  100°C for 40 min | 1:50 | Post Primary reagent (mouse IgG anti-rabbit IgG; Leica Biosystems, UK) for 20 min at 37°C and incubated with Polymer reagent (Leica Biosystems) for 20 min at 37°C. |
| **Protocol C** | Tris-EDTA buffer pH 9  digital pressure cooker  125°C for 5 min | 1:50 | biotinylated universal antibody (Dako), 20 min at room temperature; streptavidin-peroxidase (Dako) for 20 min at room temperature. |
| **Protocol C1** | Tris-EDTA buffer pH 9  digital pressure cooker  125°C for 5 min |  | Post Primary reagent (mouse IgG anti-rabbit IgG; Leica Biosystems, UK) for 20 min at 37°C and incubated with Polymer reagent (Leica Biosystems) for 20 min at 37°C. |
| **Protocol D** | citrate buffer (pH 6) digital pressure cooker 125°C for 5 min | 1:50 | biotinylated universal antibody (Dako), 20 min at room temperature; streptavidin-peroxidase (Dako) for 20 min at room temperature. |
| **Protocol D1** | citrate buffer (pH 6) digital pressure cooker 125°C for 5 min | 1:50 | Post Primary reagent (mouse IgG anti-rabbit IgG; Leica Biosystems, UK) for 20 min at 37°C and incubated with Polymer reagent (Leica Biosystems) for 20 min at 37°C. |
| **Fifth round** |  |  |  |
| **Protocol A** | Tris-EDTA buffer pH 9  digital pressure cooker  100°C for 40 min | 1:50 | Post Primary reagent (mouse IgG anti-rabbit IgG; Leica Biosystems, UK) for 20 min at 37°C and incubated with Polymer reagent (Leica Biosystems) for 20 min at 37°C. |
| **Protocol B** | Tris-EDTA buffer pH 9  digital pressure cooker  100°C for 40 min | 1:100 | Post Primary reagent (mouse IgG anti-rabbit IgG; Leica Biosystems, UK) for 20 min at 37°C and incubated with Polymer reagent (Leica Biosystems) for 20 min at 37°C. |
| **Protocol C** | Tris-EDTA buffer pH 9  digital pressure cooker  100°C for 40 min | 1:200 | Post Primary reagent (mouse IgG anti-rabbit IgG; Leica Biosystems, UK) for 20 min at 37°C and incubated with Polymer reagent (Leica Biosystems) for 20 min at 37°C. |
| **Protocol D** | Tris-EDTA buffer pH 9  digital pressure cooker  100°C for 40 min | 1:300 | Post Primary reagent (mouse IgG anti-rabbit IgG; Leica Biosystems, UK) for 20 min at 37°C and incubated with Polymer reagent (Leica Biosystems) for 20 min at 37°C. |
