# Supplementary material for: Porcine circovirus type 3: immunohistochemical detection in lesions of naturally affected piglets
Source: Front Vet Sci. 2023 May 4;10:1174718. doi: 10.3389/fvets.2023.1174718 (PMC10192697; doi:10.3389/fvets.2023.1174718)
Supplement: Supplementary file 2 [file Data_Sheet_1.PDF]

```
                    *        20         *        40         *
MW089612 : MRQRAIFRRRPRPRRRRRHRRRYARRRLFIRRPTAGTYYTKKYSTMNVIS :  50
MK033215 : ..H...............N............................... :  50
MW089613 : ..N............................................... :  50
MT497513 : ..H............................................... :  50
MN310686 : ..H............................................... :  50
MN714693 : ..H............................................... :  50
MN714692 : ..H............................................... :  50
MN714691 : ..H............................................... :  50
MK562414 : ..H............................................... :  50
MK033212 : ..H............................................... :  50
MG870100 : ..H............................................... :  50
MH184559 : ..H............................................... :  50
MF631811 : ..H............................................... :  50
MZ995502 : ..H............................................... :  50
MW089590 : ..H............................................... :  50
MG934324 : ..H............................................... :  50
MF460444 : ..H............................................... :  50
MK562416 : ..H............................................... :  50
ON184741 : ..H............................................... :  50
MW089617 : ..H............................................... :  50
MT769308 : ..H............................................... :  50
MF460442 : ..H............................................... :  50
MN714694 : -------------------------------M.................. :  19
MK562417 : ..H....................V.......................... :  50
MH184561 : ..H............................................... :  50
MT497516 : ----------------.................................. :  34
MN725087 : ..H.......S............V.......................... :  50
MK744570 : ..H...............Q............................... :  50
MT130464 : ..H............................................... :  50
MK789682 : ..H............................................... :  50
MK562412 : ..H............................................... :  50
MK033239 : ..H............................................... :  50
MK033216 : ..H............................................... :  50
MK033207 : ..H............................................... :  50
MH636587 : ..H............................................... :  50
MG770387 : ..H............................................... :  50
MG934333 : ..H............................................... :  50
MG934326 : ..H............................................... :  50
MG934323 : ..H............................................... :  50
MG934295 : ..H............................................... :  50
MH184548 : ..H............................................... :  50
MF631804 : ..H............................................... :  50
MG650179 : ..H............................................... :  50
MF374971 : ..H............................................... :  50
MF589129 : ..H............................................... :  50
MF589124 : ..H............................................... :  50
MW089547 : ..H............................................... :  50
MW089523 : ..H............................................... :  50
MW089510 : ..H............................................... :  50
MW089509 : ..H............................................... :  50
MW089504 : ..H............................................... :  50
MN703751 : ..H............................................... :  50
MN703739 : ..H............................................... :  50
MN517986 : ..H............................................... :  50
```

```
MK744590 : ..H...............Q....V.......................... :  50
MK744575 : ..H...............Q....V.......................... :  50
MK744564 : ..H...............Q....V.......................... :  50
MK744543 : ..H...............Q....V.......................... :  50
MK744576 : ..H.T.............Q....V.......................... :  50
MK033218 : ..H.............Q................................. :  50
MK033232 : ..HI.............................................. :  50
MG934303 : ..H............................................... :  50
MK744573 : ..H.T............CQ....V.......................... :  50
MH900458 : ..H....................V.......................... :  50
MH636590 : ..H....................V.......................... :  50
MF374972 : ..H....................V.......................... :  50
MW089503 : ..H....................V.......................... :  50
MK744588 : ..H............................................... :  50
MK744549 : ..H............................................... :  50
MK744546 : ..H............................................... :  50
MK744545 : ..H............................................... :  50
MK562413 : ..H............................................... :  50
MK185654 : ..H............................................... :  50
MK280765 : ..H............................................... :  50
MH900463 : ..H............................................... :  50
MH900462 : ..H............................................... :  50
MG934298 : ..H............................................... :  50
MG934289 : ..H............................................... :  50
MF589133 : ..H............................................... :  50
MF589112 : ..H............................................... :  50
MZ995500 : ..H............................................... :  50
MW089606 : ..H............................................... :  50
MW089593 : ..H............................................... :  50
MW089581 : ..H............................................... :  50
MW089576 : ..H............................................... :  50
MW089575 : ..H............................................... :  50
MW089567 : ..H............................................... :  50
MW089534 : ..H............................................... :  50
MW089530 : ..H............................................... :  50
MW089529 : ..H............................................... :  50
MN703758 : ..H............................................... :  50
MN703757 : ..H............................................... :  50
MN703756 : ..H............................................... :  50
MN703753 : ..H............................................... :  50
MN703750 : ..H............................................... :  50
MN703749 : ..H............................................... :  50
MN703748 : ..H............................................... :  50
MN703744 : ..H............................................... :  50
MN703742 : ..H............................................... :  50
MN703733 : ..H............................................... :  50

                    60         *        80         *       100
MW089612 : VGTPQNNKPWHANHFITRLNEWETAITFEYYKILKMKVTLSPVISPAQQT : 100
MK033215 : .................................................. : 100
MW089613 : .................................................. : 100
MT497513 : .................................................. : 100
MN310686 : .................................................. : 100
MN714693 : .................................................. : 100
```

```
MN714692 : .................................................. : 100
MN714691 : .................................................. : 100
MK562414 : .................................................. : 100
MK033212 : .................................................. : 100
MG870100 : .................................................. : 100
MH184559 : .................................................. : 100
MF631811 : .................................................. : 100
MZ995502 : .................................................. : 100
MW089590 : .................................................. : 100
MG934324 : .................................................. : 100
MF460444 : .................................................. : 100
MK562416 : .................................................. : 100
ON184741 : .................................................. : 100
MW089617 : .................................................. : 100
MT769308 : .................................................. : 100
MF460442 : .................................................. : 100
MN714694 : .................................................. :  69
MK562417 : .................................................. : 100
MH184561 : .................................................. : 100
MT497516 : .................................................. :  84
MN725087 : .................................................. : 100
MK744570 : .................................................. : 100
MT130464 : .................................................. : 100
MK789682 : .................................................. : 100
MK562412 : .................................................. : 100
MK033239 : .................................................. : 100
MK033216 : .................................................. : 100
MK033207 : .................................................. : 100
MH636587 : .................................................. : 100
MG770387 : .................................................. : 100
MG934333 : .................................................. : 100
MG934326 : .................................................. : 100
MG934323 : .................................................. : 100
MG934295 : .................................................. : 100
MH184548 : .................................................. : 100
MF631804 : .................................................. : 100
MG650179 : .................................................. : 100
MF374971 : .................................................. : 100
MF589129 : .................................................. : 100
MF589124 : .................................................. : 100
MW089547 : .................................................. : 100
MW089523 : .................................................. : 100
MW089510 : .................................................. : 100
MW089509 : .................................................. : 100
MW089504 : .................................................. : 100
MN703751 : .................................................. : 100
MN703739 : .................................................. : 100
MN517986 : .................................................. : 100
MK744590 : .................................................. : 100
MK744575 : .................................................. : 100
MK744564 : .................................................. : 100
MK744543 : .................................................. : 100
MK744576 : .................................................. : 100
MK033218 : .................................................. : 100
MK033232 : .................................................. : 100
```

MG934303 : .... : 100
MK744573 : .... : 100
MH900458 : .... : 100
MH636590 : .... : 100
MF374972 : .... : 100
MW089503 : .... : 100
MK744588 : .... : 100
MK744549 : .... : 100
MK744546 : .... : 100
MK744545 : .... : 100
MK562413 : .... : 100
MK185654 : .... : 100
MK280765 : .... : 100
MH900463 : .... : 100
MH900462 : .... : 100
MG934298 : .... : 100
MG934289 : .... : 100
MF589133 : .... : 100
MF589112 : .... : 100
MZ995500 : .... : 100
MW089606 : .... : 100
MW089593 : .... : 100
MW089581 : .... : 100
MW089576 : .... : 100
MW089575 : .... : 100
MW089567 : .... : 100
MW089534 : .... : 100
MW089530 : .... : 100
MW089529 : .... : 100
MN703758 : .... : 100
MN703757 : .... : 100
MN703756 : .... : 100
MN703753 : .... : 100
MN703750 : .... : 100
MN703749 : .... : 100
MN703748 : .... : 100
MN703744 : .... : 100
MN703742 : .... : 100
MN703733 : .... : 100

* 120 * 140 *
MW089612 : KTMFGHTAIDLDGAWTTNTWLQD**DPYAESSTRKVMTSKK**KHSRYFTPKPL : 150
MK033215 : .... : 150
MW089613 : .... : 150
MT497513 : .... : 150
MN310686 : .... : 150
MN714693 : .... : 150
MN714692 : .... : 150
MN714691 : .... : 150
MK562414 : .... : 150
MK033212 : .... : 150
MG870100 : .... : 150
MH184559 : .... : 150
MF631811 : .... : 150

```
MZ995502 : .................................................. : 150
MW089590 : .................................................. : 150
MG934324 : .................................................. : 150
MF460444 : .................................................. : 150
MK562416 : .................................................. : 150
ON184741 : .................................................. : 150
MW089617 : .................................................. : 150
MT769308 : .................................................. : 150
MF460442 : .................................................. : 150
MN714694 : .................................................. : 119
MK562417 : .................................................. : 150
MH184561 : .................................................. : 150
MT497516 : .................................................. : 134
MN725087 : .................................................. : 150
MK744570 : .................................................. : 150
MT130464 : .................................................. : 150
MK789682 : .................................................. : 150
MK562412 : .................................................. : 150
MK033239 : .................................................. : 150
MK033216 : .................................................. : 150
MK033207 : .................................................. : 150
MH636587 : .................................................. : 150
MG770387 : .................................................. : 150
MG934333 : .................................................. : 150
MG934326 : .................................................. : 150
MG934323 : .................................................. : 150
MG934295 : .................................................. : 150
MH184548 : .................................................. : 150
MF631804 : .................................................. : 150
MG650179 : .................................................. : 150
MF374971 : .................................................. : 150
MF589129 : .................................................. : 150
MF589124 : .................................................. : 150
MW089547 : .................................................. : 150
MW089523 : .................................................. : 150
MW089510 : .................................................. : 150
MW089509 : .................................................. : 150
MW089504 : .................................................. : 150
MN703751 : .................................................. : 150
MN703739 : .................................................. : 150
MN517986 : .................................................. : 150
MK744590 : .................................................. : 150
MK744575 : .................................................. : 150
MK744564 : .................................................. : 150
MK744543 : .................................................. : 150
MK744576 : .................................................. : 150
MK033218 : .................................................. : 150
MK033232 : .................................................. : 150
MG934303 : .................................................. : 150
MK744573 : .................................................. : 150
MH900458 : .................................................. : 150
MH636590 : .................................................. : 150
MF374972 : .................................................. : 150
MW089503 : .................................................. : 150
MK744588 : .................................................. : 150
```

MK744549 : .... : 150
MK744546 : .... : 150
MK744545 : .... : 150
MK562413 : .... : 150
MK185654 : .... : 150
MK280765 : .... : 150
MH900463 : .... : 150
MH900462 : .... : 150
MG934298 : .... : 150
MG934289 : .... : 150
MF589133 : .... : 150
MF589112 : .... : 150
MZ995500 : .... : 150
MW089606 : .... : 150
MW089593 : .... : 150
MW089581 : .... : 150
MW089576 : .... : 150
MW089575 : .... : 150
MW089567 : .... : 150
MW089534 : .... : 150
MW089530 : .... : 150
MW089529 : .... : 150
MN703758 : .... : 150
MN703757 : .... : 150
MN703756 : .... : 150
MN703753 : .... : 150
MN703750 : .... : 150
MN703749 : .... : 150
MN703748 : .... : 150
MN703744 : .... : 150
MN703742 : .... : 150
MN703733 : .... : 150

160 * 180 * 200
MW089612 : LAGTTSAHPGQSLFFFSRPTPWLNTYDPTVQWGALLWSIY**VPEKTGMTDF** : 200
MK033215 : .... : 200
MW089613 : .... : 200
MT497513 : .... : 200
MN310686 : .... : 200
MN714693 : .... : 200
MN714692 : .... : 200
MN714691 : .... : 200
MK562414 : .... : 200
MK033212 : .... : 200
MG870100 : .... : 200
MH184559 : .... : 200
MF631811 : .... : 200
MZ995502 : .... : 200
MW089590 : .... : 200
MG934324 : .... : 200
MF460444 : .... : 200
MK562416 : .... : 200
ON184741 : .... : 200
MW089617 : .... : 200

```
MT769308 : .................................................. : 200
MF460442 : .................................................. : 200
MN714694 : .................................................. : 169
MK562417 : .................................................. : 200
MH184561 : ......S........................................... : 200
MT497516 : .................................................. : 184
MN725087 : .................................................. : 200
MK744570 : .................................................. : 200
MT130464 : .................................................. : 200
MK789682 : .................................................. : 200
MK562412 : .................................................. : 200
MK033239 : .................................................. : 200
MK033216 : .................................................. : 200
MK033207 : .................................................. : 200
MH636587 : .................................................. : 200
MG770387 : .................................................. : 200
MG934333 : .................................................. : 200
MG934326 : .................................................. : 200
MG934323 : .................................................. : 200
MG934295 : .................................................. : 200
MH184548 : .................................................. : 200
MF631804 : .................................................. : 200
MG650179 : .................................................. : 200
MF374971 : .................................................. : 200
MF589129 : .................................................. : 200
MF589124 : .................................................. : 200
MW089547 : .................................................. : 200
MW089523 : .................................................. : 200
MW089510 : .................................................. : 200
MW089509 : .................................................. : 200
MW089504 : .................................................. : 200
MN703751 : .................................................. : 200
MN703739 : .................................................. : 200
MN517986 : .................................................. : 200
MK744590 : .................................................. : 200
MK744575 : .................................................. : 200
MK744564 : .................................................. : 200
MK744543 : .................................................. : 200
MK744576 : .................................................. : 200
MK033218 : .................................................. : 200
MK033232 : .................................................. : 200
MG934303 : .................................................. : 200
MK744573 : .................................................. : 200
MH900458 : .................................................. : 200
MH636590 : .................................................. : 200
MF374972 : .................................................. : 200
MW089503 : .................................................. : 200
MK744588 : .................................................. : 200
MK744549 : .................................................. : 200
MK744546 : .................................................. : 200
MK744545 : .................................................. : 200
MK562413 : .................................................. : 200
MK185654 : .................................................. : 200
MK280765 : .................................................. : 200
MH900463 : .................................................. : 200
```

```
MH900462 : .................................................. : 200
MG934298 : .................................................. : 200
MG934289 : .................................................. : 200
MF589133 : .................................................. : 200
MF589112 : .................................................. : 200
MZ995500 : .................................................. : 200
MW089606 : .................................................. : 200
MW089593 : .................................................. : 200
MW089581 : .................................................. : 200
MW089576 : .................................................. : 200
MW089575 : .................................................. : 200
MW089567 : .................................................. : 200
MW089534 : .................................................. : 200
MW089530 : .................................................. : 200
MW089529 : .................................................. : 200
MN703758 : .................................................. : 200
MN703757 : .................................................. : 200
MN703756 : .................................................. : 200
MN703753 : .................................................. : 200
MN703750 : .................................................. : 200
MN703749 : .................................................. : 200
MN703748 : .................................................. : 200
MN703744 : .................................................. : 200
MN703742 : .................................................. : 200
MN703733 : .................................................. : 200


                    *
MW089612 : YGTKEVWIRYKSVL : 214
MK033215 : .............. : 214
MW089613 : .............. : 214
MT497513 : .............. : 214
MN310686 : .............. : 214
MN714693 : .............. : 214
MN714692 : .............. : 214
MN714691 : .............. : 214
MK562414 : .............. : 214
MK033212 : .............. : 214
MG870100 : .............. : 214
MH184559 : .............. : 214
MF631811 : .............. : 214
MZ995502 : .............. : 214
MW089590 : .............. : 214
MG934324 : .............. : 214
MF460444 : .............. : 214
MK562416 : .............. : 214
ON184741 : .............. : 214
MW089617 : .............. : 214
MT769308 : .............. : 214
MF460442 : .............. : 214
MN714694 : .............. : 183
MK562417 : .............. : 214
MH184561 : .............. : 214
MT497516 : .............. : 198
MN725087 : .............. : 214
```

```
MK744570 : .............. : 214
MT130464 : .............. : 214
MK789682 : .............. : 214
MK562412 : .............. : 214
MK033239 : .............. : 214
MK033216 : .............. : 214
MK033207 : .............. : 214
MH636587 : .............. : 214
MG770387 : .............. : 214
MG934333 : .............. : 214
MG934326 : .............. : 214
MG934323 : .............. : 214
MG934295 : .............. : 214
MH184548 : .............. : 214
MF631804 : .............. : 214
MG650179 : .............. : 214
MF374971 : .............. : 214
MF589129 : .............. : 214
MF589124 : .............. : 214
MW089547 : .............. : 214
MW089523 : .............. : 214
MW089510 : .............. : 214
MW089509 : .............. : 214
MW089504 : .............. : 214
MN703751 : .............. : 214
MN703739 : .............. : 214
MN517986 : .............. : 214
MK744590 : .............. : 214
MK744575 : .............. : 214
MK744564 : .............. : 214
MK744543 : .............. : 214
MK744576 : .............. : 214
MK033218 : .............. : 214
MK033232 : .............. : 214
MG934303 : .............. : 214
MK744573 : .............. : 214
MH900458 : .............. : 214
MH636590 : .............. : 214
MF374972 : .............. : 214
MW089503 : .............. : 214
MK744588 : .............. : 214
MK744549 : .............. : 214
MK744546 : .............. : 214
MK744545 : .............. : 214
MK562413 : .............. : 214
MK185654 : .............. : 214
MK280765 : .............. : 214
MH900463 : .............. : 214
MH900462 : .............. : 214
MG934298 : .............. : 214
MG934289 : .............. : 214
MF589133 : .............. : 214
MF589112 : .............. : 214
MZ995500 : .............. : 214
MW089606 : .............. : 214
```

```
MW089593 : .............. : 214
MW089581 : .............. : 214
MW089576 : .............. : 214
MW089575 : .............. : 214
MW089567 : .............. : 214
MW089534 : .............. : 214
MW089530 : .............. : 214
MW089529 : .............. : 214
MN703758 : .............. : 214
MN703757 : .............. : 214
MN703756 : .............. : 214
MN703753 : .............. : 214
MN703750 : .............. : 214
MN703749 : .............. : 214
MN703748 : .............. : 214
MN703744 : .............. : 214
MN703742 : .............. : 214
MN703733 : .............. : 214
```
