# Supplementary figures and images for: Porcine circovirus type 3: immunohistochemical detection in lesions of naturally affected piglets
Source: Front Vet Sci. 2023 May 4;10:1174718. doi: 10.3389/fvets.2023.1174718 (PMC10192697; doi:10.3389/fvets.2023.1174718)

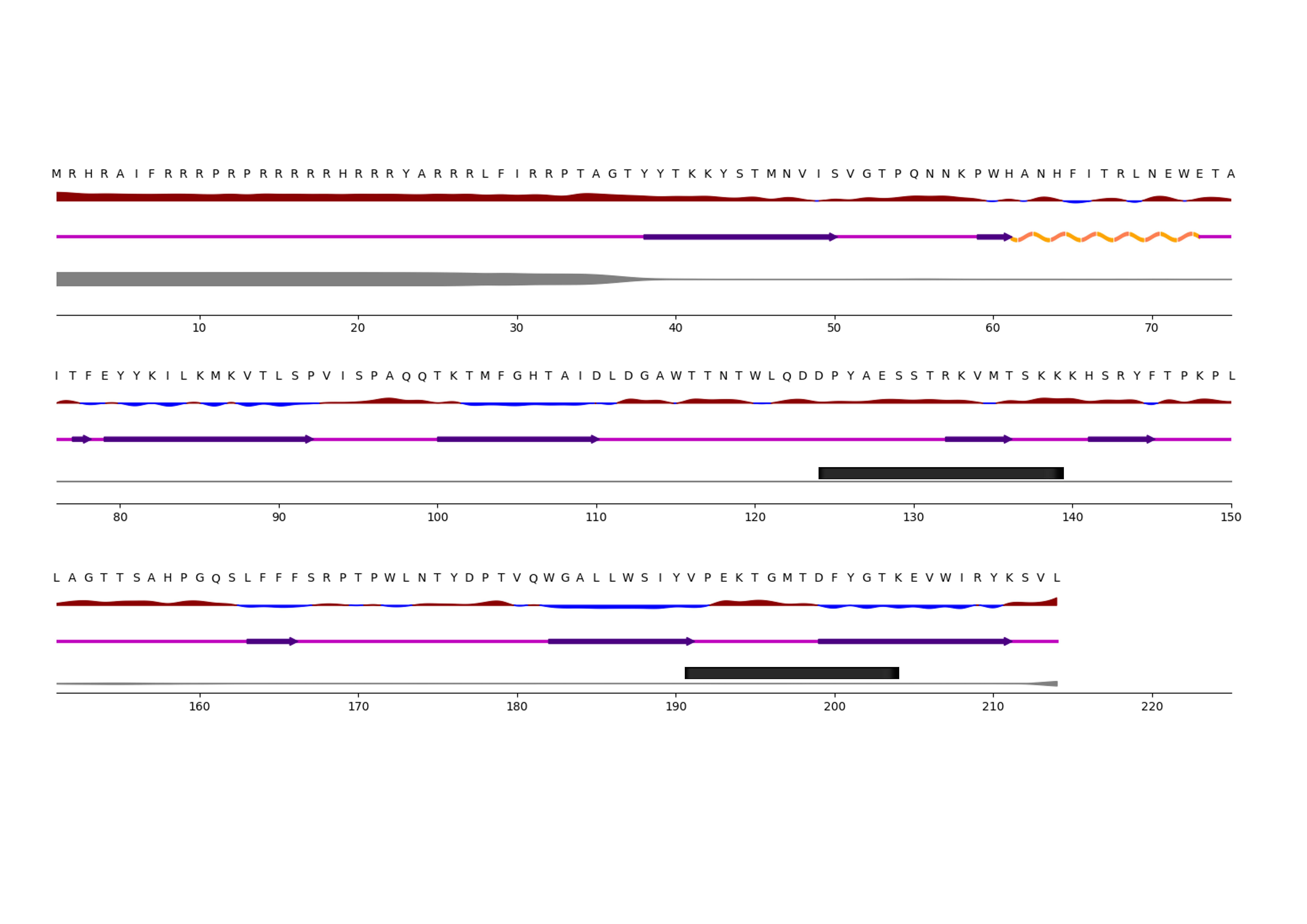

Supplement: Supplementary file 3 [file Image_1.TIF]
